# Crystal structure of 2,3-diphenyl-2,3-di­hydro-4*H*-1,3-benzo­thia­zin-4-one 1-oxide

**DOI:** 10.1107/S2056989017010313

**Published:** 2017-07-17

**Authors:** Hemant P. Yennawar, Ryan Fox, Quentin J. Moyer, Ziwei Yang, Lee J. Silverberg

**Affiliations:** a Department of Biochemistry and Molecular Biology, Pennsylvania State University, University Park, PA 16802, USA; b Pennsylvania State University, Schuylkill Campus, 200 University Drive, Schuylkill Haven, PA 17972, USA

**Keywords:** crystal structure, benzo­thia­zine, screw-boat pucker, S-oxide, 1,3-benzo­thia­zin-4-one

## Abstract

In the racemic title compound, C_20_H_15_NO_2_S, the planes of the two phenyl substituents form dihedral angles of 48.97 (15) and 69.26 (15)° with that of the fused benzene ring of the parent benzo­thia­zine ring, while the heterocyclic thia­zine ring exhibits a screw-boat pucker. The O atom on the S atom of the ring is pseudo-axial on the thia­zine ring and *trans* to the 2-phenyl group. In the crystal, mol­ecules are arranged in layers in the *ac* plane, the layers being linked across *b* through inter­molecular C—H⋯O hydrogen-bonding inter­actions.

## Abstract 2

The crystal structure of the sulfoxide of 2,3-diphenyl-2,3-di­hydro-4*H*-1,3-benzo­thia­zin-4-one, which belongs to a bioactive family of compounds, exhibits a screw-boat pucker for the thia­zine ring. C—H⋯O hydrogen-bond and van der Waals inter­actions stabilize the crystal lattice.

## Chemical context   

The 2,3-di­hydro-4*H*-1,3-benzo­thia­zin-4-one scaffold has shown a wide range of bioactivity, including anti­tumor (Li *et al.*, 2012 ▸; Wang *et al.*, 2015 ▸; Kamel *et al.*, 2010 ▸; Nofal *et al.*, 2014 ▸), anti­microbial (Popiolek *et al.*, 2016 ▸; Mandour *et al.*, 2007 ▸), anti­malarial (Mei *et al.*, 2013 ▸), HIV–RT inhibition (Jeng *et al.*, 2015 ▸; Hou *et al.*, 2016 ▸) and cyclo­oxygenase COX-2 enzyme inhibition (Zarghi *et al.*, 2009 ▸). The *S*-oxides of these compounds have been little studied (a search found fewer than 50), despite the evidence of enhanced activity in the similar 2,3,5,6-tetra­hydro-4*H*-1,3-thia­zin-4-ones (Surrey *et al.*, 1958 ▸; Surrey, 1963*a* ▸,*b* ▸) and 1,3-thia­zolidin-4-ones (Gududuru *et al.*, 2004 ▸). Also of potential inter­est is the tri­phenyl­tin chloride adduct, which may have enhanced anti­fungal activity (Eng *et al.*, 1996 ▸).
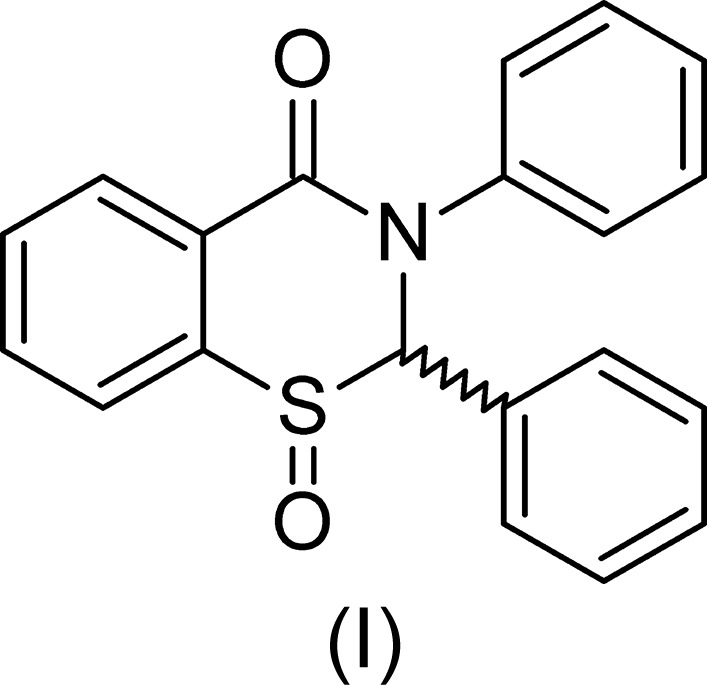


Recently, we reported the crystal structures of 2,3-diphenyl-2,3,5,6-tetra­hydro-4*H*-1,3-thia­zine-4-one 1-oxide (Yennawar, Yang & Silverberg, 2016 ▸) and the 1:1 adduct of tri­phenyl­tin chloride and 2,3-diphenyl-2,3,5,6-tetra­hydro-4*H*-1,3-thia­zin-4-one (Yennawar, Fox & Silverberg, 2016 ▸). Attempts to prepare the tri­phenyl­tin chloride adduct of 2,3-diphenyl-2,3-di­hydro-4*H*-1,3-benzo­thia­zin-4-one instead produced the sulfoxide 2,3-diphenyl-2,3-di­hydro-4*H*-1,3-benzo­thia­zin-4-one 1-oxide on two separate occasions. The sulfoxide was also intentionally prepared by oxidation of 2,3-diphenyl-2,3-di­hydro-4*H*-1,3-benzo­thia­zin-4-one with Oxone^®^. It has not yet been determined how the sulfoxide formed in the tin reaction, but Bourgoin-Legay & Boudet (1969 ▸) have reported the air oxidation of 2-alkyl-4*H*-1,3-benzo­thia­zines to give the sulfoxides, although the analogous 2-aryl compounds were less prone to air oxidation.

In this article, we report the crystal structure of the product from one of the reactions using tin, the title compound, namely 2,3-diphenyl-2,3-di­hydro-4*H*-1,3-benzo­thia­zin-4-one 1-oxide, (I). To the best of our knowledge, this is the first reported crystal structure of an *S*-oxide of a 2,3-di­hydro-4*H*-1,3-benzo­thia­zin-4-one.

## Structural commentary   

**Figure 1:**
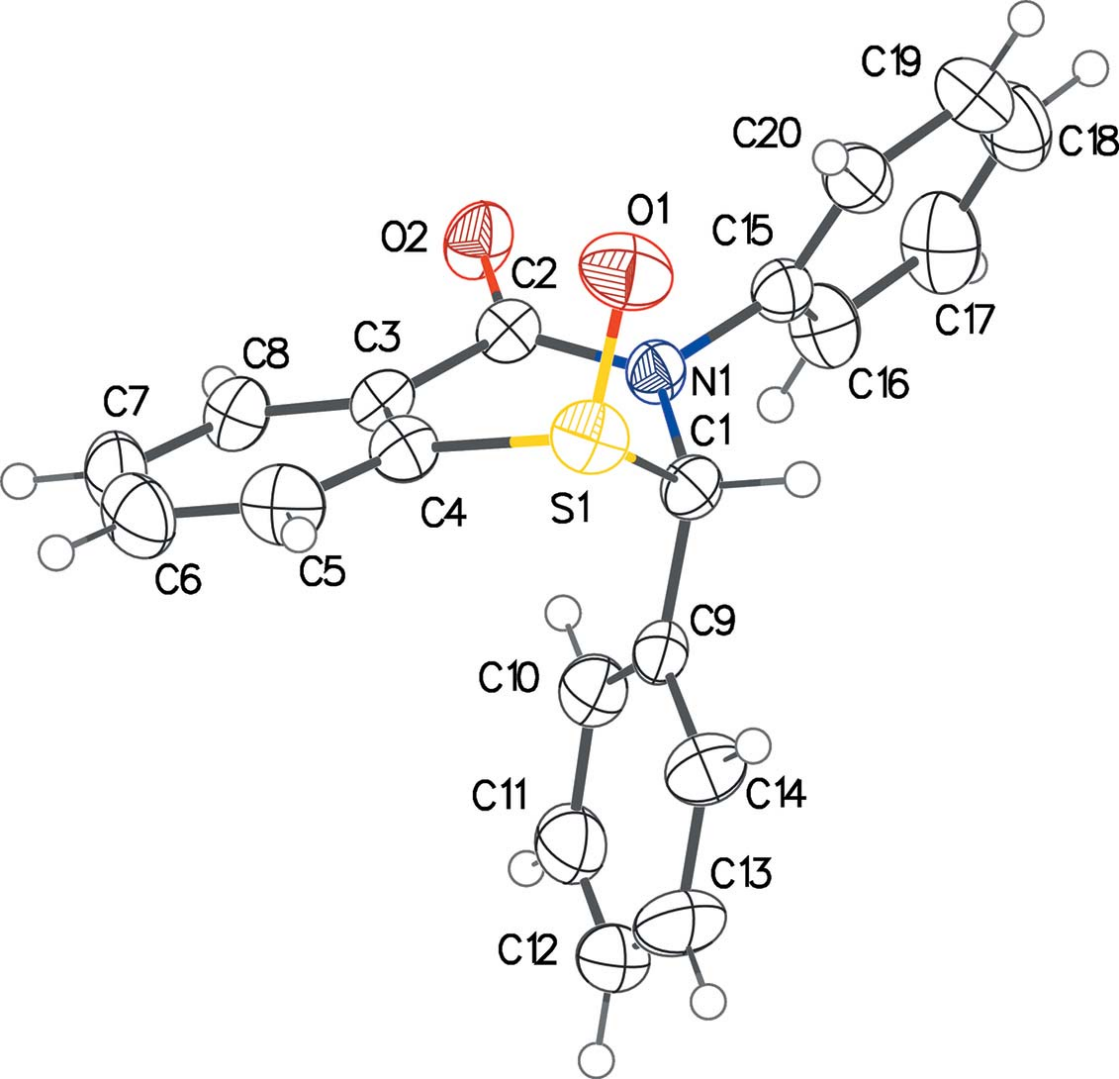
The mol­ecular structure of the title compound, with displacement ellipsoids drawn at the 50% probability level.

In the title racemic compound, the planes of the two phenyl substituents form dihedral angles of 48.97 (15) and 69.26 (15)° with that of the fused benzene ring of the parent benzo­thia­zine system (Fig. 1 ▸). The O atom on the S atom is pseudo-axial and *trans* to the 2-phenyl ring, just as in 2,3-diphenyl-2,3,5,6-tetra­hydro-4*H*-1,3-thia­zin-4-one 1-oxide (Yennawar, Yang & Silverberg, 2016 ▸). The thia­zine ring has a screw-boat conformation, with a puckering amplitude of 0.686 (2) Å and θ = 65.6 (2)° (Cremer & Pople, 1975 ▸). The thia­zine ring in 2,3-diphenyl-2,3,5,6-tetra­hydro-4*H*-1,3-thia­zin-4-one 1-oxide (Yennawar, Yang & Silverberg, 2016 ▸) was in an envelope conformation. The overall mol­ecular configuration is quite similar to the structure of 2,3-diphenyl-2,3-di­hydro-4*H*-1,3-benzo­thia­zin-4-one (Yennawar *et al.*, 2014 ▸).

## Supra­molecular features   

**Figure 2:**
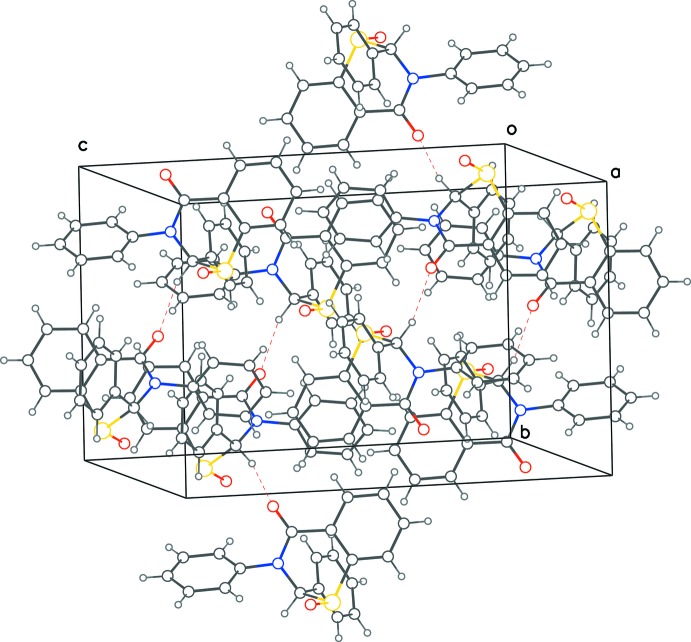
Crystal packing diagram showing C—H⋯O contacts as dotted red lines between mol­ecules of (I) which form chains along the *b*-axis direction.

The crystal lattice has layers of mol­ecules comprising alternating enanti­omers, extending along the *a*-axis direction and lying in the *ac* plane. The layers are linked across the *b*-cell direction through inter­molecular C1—H⋯O2^i^ hydrogen bonds (Fig. 2 ▸, Table 1 ▸) between mol­ecules of the same chirality [symmetry code: (i) −*x* + 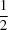, *y* − 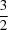, −*z* + 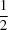]. While C—H⋯O inter­actions are also present in our two earlier structures (Yennawar *et al.*, 2014 ▸; Yennawar, Yang & Silverberg, 2016 ▸), the differences in either the donor C or acceptor O atoms make them unique in each case. In the present structure, the chiral C atom donates the proton to the O atom at position 4 (⋯O—C) of the thia­zine ring, while in our 2016 ▸ structure, the acceptor O atom was the one at position 1 (⋯O—S). In the 2014 ▸ structure, the two benzene-ring C atoms are the donors to the only O atom (⋯O—C) on the thia­zine ring.

## Database survey   

A literature search found no prior reports of a crystal structure of an *S*-oxide of a 2,3-di­hydro-4*H*-1,3-benzo­thia­zin-4-one. We have previously reported the crystal structures of 2,3-diphenyl-2,3,5,6-tetra­hydro-4*H*-1,3-thia­zin-4-one 1-oxide (Yennawar, Yang & Silverberg, 2016 ▸) and 2,3-diphenyl-2,3-di­hydro-4*H*-1,3-benzo­thia­zin-4-one (Yennawar *et al.*, 2014 ▸).

## Synthesis and crystallization   

A 2 ml reactivial with a stir bar was charged with 0.1004 g of 2,3-diphenyl-2,3-di­hydro-4*H*-1,3-benzo­thia­zin-4-one and 0.95 ml of acetone and stirred. The benzo­thia­zinone did not fully dissolve. A 10 ml round-bottomed flask was charged with 0.1212 g of tri­phenyl­tin chloride and 2.0 ml of acetone and stirred. The contents of the 2 ml vial were added to the 10 ml flask and the vial was rinsed with an additional 0.5 ml of acetone, giving a clear solution, which was stirred for 2 h and then allowed to stand without stirring for 3 d. The solution was filtered through Celite and then concentrated under vacuum, giving a white solid. The solid was recrystallized from cyclo­hexane to give a yellow solid (yield 0.0755 g, 72%). Crystals suitable for X-ray analysis were obtained by slow evaporation from an acetone solution.

## Refinement   

Crystal data, data collection and structure refinement details are summarized in Table 2 ▸. The H atoms were placed geometrically and allowed to ride on their parent C atoms during refinement, with C—H distances of 0.98 (methine) or 0.93 Å (aromatic) and with *U*_iso_(H) = 1.2*U*_eq_(C). Although of no particular significance in this racemic compound, the enanti­omer chosen was the C1(*S*) one.

## Supplementary Material

Crystal structure: contains datablock(s) I. DOI: 10.1107/S2056989017010313/zs2384sup1.cif

Structure factors: contains datablock(s) I. DOI: 10.1107/S2056989017010313/zs2384Isup2.hkl

Supporting information file. DOI: 10.1107/S2056989017010313/zs2384Isup3.mol

Supporting information file. DOI: 10.1107/S2056989017010313/zs2384Isup4.cml

CCDC reference: 1561660

Additional supporting information:  crystallographic information; 3D view; checkCIF report

## Figures and Tables

**Table 1:** Hydrogen-bond geometry (Å, °)

*D*—H⋯*A*	*D*—H	H⋯*A*	*D*⋯*A*	*D*—H⋯*A*
C1—H1⋯O2^i^	0.98	2.31	3.240 (3)	157

Symmetry code: (i) 
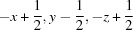
.

**Table 2:** Experimental details

Crystal data	
Chemical formula	C_20_H_15_NO_2_S
*M*_r_	333.39
Crystal system, space group	Monoclinic, *P*2_1_/*n*
Temperature (K)	298
*a*, *b*, *c* (Å)	9.1505 (16), 11.2712 (19), 16.379 (3)
β (°)	103.997 (6)
*V* (Å^3^)	1639.2 (5)
*Z*	4
Radiation type	Mo *K*α
μ (mm^−1^)	0.21
Crystal size (mm)	0.20 × 0.16 × 0.14

Data collection	
Diffractometer	Bruker CCD area detector
Absorption correction	Multi-scan (*SADABS*; Bruker, 2001 ▸)
*T*_min_, *T*_max_	0.790, 0.9
No. of measured, independent and observed [*I* > 2σ(*I*)] reflections	12730, 4036, 3701
*R*_int_	0.025
(sin θ/λ)_max_ (Å^−1^)	0.668

Refinement	
*R*[*F* ^2^ > 2σ(*F* ^2^)], *wR*(*F* ^2^), *S*	0.090, 0.231, 1.65
No. of reflections	4036
No. of parameters	217
H-atom treatment	H-atom parameters constrained
Δρ_max_, Δρ_min_ (e Å^−3^)	0.61, −0.34

Computer programs: *SMART* (Bruker, 2001 ▸), *SAINT* (Bruker, 2001 ▸), *olex2.solve* (Bourhis *et al.*, 2015 ▸), *SHELXL97* (Sheldrick, 2008 ▸) and *OLEX2* (Dolomanov *et al.*, 2009 ▸).

## supplementary crystallographic information

### Crystal data

**Table:**

C_20_H_15_NO_2_S	*F*(000) = 696
*M**_r_* = 333.39	*D*_x_ = 1.351 Mg m^−^^3^
Monoclinic, *P*2_1_/*n*	Mo *K*α radiation, λ = 0.71073 Å
*a* = 9.1505 (16) Å	Cell parameters from 975 reflections
*b* = 11.2712 (19) Å	θ = 2.9–28.1°
*c* = 16.379 (3) Å	µ = 0.21 mm^−^^1^
β = 103.997 (6)°	*T* = 298 K
*V* = 1639.2 (5) Å^3^	Block, colorless
*Z* = 4	0.20 × 0.16 × 0.14 mm

### Data collection

**Table:**

Bruker CCD area detector diffractometer	4036 independent reflections
Radiation source: fine-focus sealed tube	3701 reflections with *I* > 2σ(*I*)
Graphite monochromator	*R*_int_ = 0.025
phi and ω scans	θ_max_ = 28.4°, θ_min_ = 2.2°
Absorption correction: multi-scan (SADABS; Bruker, 2001)	*h* = −12→11
*T*_min_ = 0.790, *T*_max_ = 0.9	*k* = −14→14
12730 measured reflections	*l* = −21→21

### Refinement

**Table:**

Refinement on *F*^2^	Primary atom site location: iterative
Least-squares matrix: full	Secondary atom site location: difference Fourier map
*R*[*F*^2^ > 2σ(*F*^2^)] = 0.090	Hydrogen site location: inferred from neighbouring sites
*wR*(*F*^2^) = 0.231	H-atom parameters constrained
*S* = 1.65	*w* = 1/[σ^2^(*F*_o_^2^) + (0.1*P*)^2^] where *P* = (*F*_o_^2^ + 2*F*_c_^2^)/3
4036 reflections	(Δ/σ)_max_ < 0.001
217 parameters	Δρ_max_ = 0.61 e Å^−^^3^
0 restraints	Δρ_min_ = −0.34 e Å^−^^3^

### Special details

**Table:**

Experimental. The data collection nominally covered a full sphere of reciprocal space by a combination of 4 sets of ω scans each set at different φ and/or 2θ angles and each scan (10 s exposure) covering -0.300° degrees in ω. The crystal to detector distance was 5.82 cm.
Geometry. All esds (except the esd in the dihedral angle between two l.s. planes) are estimated using the full covariance matrix. The cell esds are taken into account individually in the estimation of esds in distances, angles and torsion angles; correlations between esds in cell parameters are only used when they are defined by crystal symmetry. An approximate (isotropic) treatment of cell esds is used for estimating esds involving l.s. planes.
Refinement. Refinement of F^2^ against ALL reflections. The weighted R-factor wR and goodness of fit S are based on F^2^, conventional R-factors R are based on F, with F set to zero for negative F^2^. The threshold expression of F^2^ > 2sigma(F^2^) is used only for calculating R-factors(gt) etc. and is not relevant to the choice of reflections for refinement. R-factors based on F^2^ are statistically about twice as large as those based on F, and R- factors based on ALL data will be even larger.

### Fractional atomic coordinates and isotropic or equivalent isotropic displacement parameters (Å^2^)

**Table:**

	*x*	*y*	*z*	*U*_iso_*/*U*_eq_	
C1	0.2072 (3)	0.6272 (2)	0.31199 (16)	0.0321 (5)	
H1	0.1959	0.5611	0.2720	0.039*	
C2	0.3510 (3)	0.8148 (2)	0.32892 (15)	0.0295 (5)	
C3	0.3394 (3)	0.8157 (2)	0.41882 (15)	0.0303 (5)	
C4	0.3293 (3)	0.7126 (2)	0.46419 (16)	0.0341 (6)	
C5	0.3268 (3)	0.7173 (3)	0.54783 (18)	0.0473 (7)	
H5	0.3222	0.6477	0.5776	0.057*	
C6	0.3311 (4)	0.8261 (3)	0.58734 (19)	0.0516 (8)	
H6	0.3298	0.8298	0.6439	0.062*	
C7	0.3373 (3)	0.9288 (3)	0.54312 (18)	0.0467 (7)	
H7	0.3384	1.0019	0.5696	0.056*	
C8	0.3420 (3)	0.9239 (2)	0.45941 (17)	0.0384 (6)	
H8	0.3470	0.9939	0.4301	0.046*	
C9	0.0503 (3)	0.6611 (2)	0.31841 (15)	0.0339 (6)	
C10	−0.0061 (3)	0.7748 (3)	0.30135 (18)	0.0434 (7)	
H10	0.0541	0.8338	0.2868	0.052*	
C11	−0.1521 (4)	0.8012 (3)	0.3059 (2)	0.0562 (9)	
H11	−0.1896	0.8776	0.2938	0.067*	
C12	−0.2407 (4)	0.7160 (4)	0.3280 (2)	0.0648 (10)	
H12	−0.3379	0.7346	0.3316	0.078*	
C13	−0.1875 (4)	0.6036 (4)	0.3447 (3)	0.0673 (10)	
H13	−0.2485	0.5458	0.3599	0.081*	
C14	−0.0414 (4)	0.5746 (3)	0.3393 (2)	0.0526 (8)	
H14	−0.0061	0.4974	0.3498	0.063*	
C15	0.3197 (3)	0.7045 (2)	0.19894 (16)	0.0320 (5)	
C16	0.2398 (4)	0.7703 (3)	0.13225 (17)	0.0446 (7)	
H16	0.1642	0.8216	0.1393	0.053*	
C17	0.2729 (5)	0.7592 (3)	0.05491 (19)	0.0574 (9)	
H17	0.2198	0.8038	0.0096	0.069*	
C18	0.3845 (4)	0.6825 (3)	0.0442 (2)	0.0582 (9)	
H18	0.4082	0.6770	−0.0077	0.070*	
C19	0.4602 (4)	0.6144 (3)	0.1105 (2)	0.0548 (8)	
H19	0.5333	0.5610	0.1030	0.066*	
C20	0.4279 (3)	0.6249 (3)	0.18879 (18)	0.0430 (7)	
H20	0.4787	0.5787	0.2338	0.052*	
N1	0.2910 (2)	0.71883 (18)	0.28149 (13)	0.0304 (5)	
O1	0.4735 (2)	0.5462 (2)	0.39909 (15)	0.0535 (6)	
O2	0.4139 (2)	0.89553 (17)	0.30137 (12)	0.0421 (5)	
S1	0.32220 (8)	0.57148 (6)	0.41423 (4)	0.0392 (3)	

### Atomic displacement parameters (Å^2^)

**Table:**

	*U*^11^	*U*^22^	*U*^33^	*U*^12^	*U*^13^	*U*^23^
C1	0.0354 (12)	0.0251 (11)	0.0372 (13)	−0.0058 (9)	0.0112 (10)	−0.0019 (10)
C2	0.0296 (11)	0.0270 (11)	0.0303 (12)	0.0005 (9)	0.0041 (9)	0.0034 (9)
C3	0.0273 (11)	0.0307 (12)	0.0309 (13)	−0.0032 (9)	0.0032 (9)	0.0014 (9)
C4	0.0332 (12)	0.0328 (12)	0.0351 (13)	0.0028 (10)	0.0059 (10)	0.0033 (10)
C5	0.0507 (16)	0.0536 (17)	0.0366 (15)	0.0039 (13)	0.0087 (12)	0.0134 (13)
C6	0.0555 (18)	0.068 (2)	0.0303 (14)	0.0079 (15)	0.0083 (13)	−0.0011 (14)
C7	0.0493 (16)	0.0513 (17)	0.0372 (15)	0.0007 (13)	0.0062 (12)	−0.0131 (13)
C8	0.0415 (14)	0.0347 (14)	0.0370 (14)	−0.0045 (10)	0.0058 (11)	−0.0013 (11)
C9	0.0342 (12)	0.0395 (14)	0.0284 (12)	−0.0097 (10)	0.0079 (10)	−0.0089 (10)
C10	0.0387 (14)	0.0467 (15)	0.0431 (15)	0.0005 (12)	0.0065 (12)	−0.0077 (13)
C11	0.0443 (17)	0.070 (2)	0.0495 (18)	0.0094 (15)	0.0029 (14)	−0.0180 (16)
C12	0.0364 (15)	0.101 (3)	0.058 (2)	−0.0051 (18)	0.0132 (14)	−0.032 (2)
C13	0.0468 (18)	0.089 (3)	0.072 (2)	−0.0270 (19)	0.0265 (17)	−0.011 (2)
C14	0.0475 (16)	0.0529 (19)	0.0606 (19)	−0.0145 (14)	0.0196 (15)	−0.0049 (15)
C15	0.0343 (12)	0.0315 (12)	0.0312 (12)	−0.0057 (9)	0.0101 (10)	−0.0017 (10)
C16	0.0587 (18)	0.0383 (14)	0.0378 (15)	0.0025 (12)	0.0136 (13)	0.0016 (12)
C17	0.085 (2)	0.0500 (18)	0.0366 (16)	−0.0038 (17)	0.0139 (15)	0.0080 (14)
C18	0.078 (2)	0.063 (2)	0.0414 (17)	−0.0122 (17)	0.0293 (17)	−0.0071 (15)
C19	0.0516 (18)	0.065 (2)	0.0541 (19)	0.0021 (15)	0.0252 (15)	−0.0110 (16)
C20	0.0379 (14)	0.0530 (17)	0.0380 (15)	0.0047 (12)	0.0089 (11)	−0.0052 (12)
N1	0.0350 (10)	0.0270 (10)	0.0305 (10)	−0.0046 (8)	0.0106 (8)	−0.0016 (8)
O1	0.0475 (12)	0.0473 (12)	0.0673 (14)	0.0153 (9)	0.0169 (10)	0.0093 (10)
O2	0.0545 (12)	0.0346 (10)	0.0385 (10)	−0.0164 (8)	0.0139 (9)	0.0009 (8)
S1	0.0453 (4)	0.0278 (4)	0.0463 (4)	0.0023 (2)	0.0145 (3)	0.0098 (3)

### Geometric parameters (Å, º)

**Table:**

C1—H1	0.9800	C10—C11	1.388 (4)
C1—C9	1.514 (3)	C11—H11	0.9300
C1—N1	1.446 (3)	C11—C12	1.361 (5)
C1—S1	1.858 (3)	C12—H12	0.9300
C2—C3	1.502 (3)	C12—C13	1.361 (6)
C2—N1	1.367 (3)	C13—H13	0.9300
C2—O2	1.220 (3)	C13—C14	1.400 (5)
C3—C4	1.394 (3)	C14—H14	0.9300
C3—C8	1.386 (3)	C15—C16	1.375 (4)
C4—C5	1.377 (4)	C15—C20	1.375 (4)
C4—S1	1.783 (3)	C15—N1	1.447 (3)
C5—H5	0.9300	C16—H16	0.9300
C5—C6	1.383 (4)	C16—C17	1.378 (4)
C6—H6	0.9300	C17—H17	0.9300
C6—C7	1.374 (4)	C17—C18	1.381 (5)
C7—H7	0.9300	C18—H18	0.9300
C7—C8	1.383 (4)	C18—C19	1.373 (5)
C8—H8	0.9300	C19—H19	0.9300
C9—C10	1.384 (4)	C19—C20	1.389 (4)
C9—C14	1.383 (4)	C20—H20	0.9300
C10—H10	0.9300	O1—S1	1.491 (2)
			
C9—C1—H1	106.9	C12—C11—H11	119.8
C9—C1—S1	111.28 (17)	C11—C12—H12	119.9
N1—C1—H1	106.9	C13—C12—C11	120.2 (3)
N1—C1—C9	115.8 (2)	C13—C12—H12	119.9
N1—C1—S1	108.68 (16)	C12—C13—H13	119.8
S1—C1—H1	106.9	C12—C13—C14	120.4 (3)
N1—C2—C3	116.7 (2)	C14—C13—H13	119.8
O2—C2—C3	120.6 (2)	C9—C14—C13	119.8 (3)
O2—C2—N1	122.7 (2)	C9—C14—H14	120.1
C4—C3—C2	123.1 (2)	C13—C14—H14	120.1
C8—C3—C2	118.6 (2)	C16—C15—N1	120.0 (2)
C8—C3—C4	118.3 (2)	C20—C15—C16	121.0 (3)
C3—C4—S1	119.9 (2)	C20—C15—N1	119.0 (2)
C5—C4—C3	121.2 (2)	C15—C16—H16	120.4
C5—C4—S1	118.9 (2)	C15—C16—C17	119.2 (3)
C4—C5—H5	120.2	C17—C16—H16	120.4
C4—C5—C6	119.6 (3)	C16—C17—H17	119.8
C6—C5—H5	120.2	C16—C17—C18	120.5 (3)
C5—C6—H6	120.0	C18—C17—H17	119.8
C7—C6—C5	120.0 (3)	C17—C18—H18	120.1
C7—C6—H6	120.0	C19—C18—C17	119.8 (3)
C6—C7—H7	119.9	C19—C18—H18	120.1
C6—C7—C8	120.3 (3)	C18—C19—H19	119.9
C8—C7—H7	119.9	C18—C19—C20	120.2 (3)
C3—C8—H8	119.7	C20—C19—H19	119.9
C7—C8—C3	120.6 (2)	C15—C20—C19	119.2 (3)
C7—C8—H8	119.7	C15—C20—H20	120.4
C10—C9—C1	122.2 (2)	C19—C20—H20	120.4
C14—C9—C1	118.8 (3)	C1—N1—C15	118.32 (19)
C14—C9—C10	119.0 (3)	C2—N1—C1	122.9 (2)
C9—C10—H10	119.9	C2—N1—C15	118.7 (2)
C9—C10—C11	120.3 (3)	C4—S1—C1	93.56 (11)
C11—C10—H10	119.9	O1—S1—C1	105.12 (12)
C10—C11—H11	119.8	O1—S1—C4	108.31 (13)
C12—C11—C10	120.4 (3)		
			
C1—C9—C10—C11	178.3 (2)	C15—C16—C17—C18	0.4 (5)
C1—C9—C14—C13	−179.3 (3)	C16—C15—C20—C19	2.4 (4)
C2—C3—C4—C5	176.6 (2)	C16—C15—N1—C1	105.0 (3)
C2—C3—C4—S1	−2.5 (3)	C16—C15—N1—C2	−78.4 (3)
C2—C3—C8—C7	−177.6 (2)	C16—C17—C18—C19	1.7 (5)
C3—C2—N1—C1	5.9 (3)	C17—C18—C19—C20	−1.9 (5)
C3—C2—N1—C15	−170.5 (2)	C18—C19—C20—C15	−0.2 (5)
C3—C4—C5—C6	1.3 (4)	C20—C15—C16—C17	−2.5 (4)
C3—C4—S1—C1	−36.3 (2)	C20—C15—N1—C1	−75.2 (3)
C3—C4—S1—O1	70.9 (2)	C20—C15—N1—C2	101.3 (3)
C4—C3—C8—C7	1.1 (4)	N1—C1—C9—C10	−4.0 (3)
C4—C5—C6—C7	0.3 (5)	N1—C1—C9—C14	173.9 (2)
C5—C4—S1—C1	144.5 (2)	N1—C1—S1—C4	60.72 (18)
C5—C4—S1—O1	−108.3 (2)	N1—C1—S1—O1	−49.3 (2)
C5—C6—C7—C8	−1.2 (5)	N1—C2—C3—C4	26.3 (3)
C6—C7—C8—C3	0.5 (4)	N1—C2—C3—C8	−155.1 (2)
C8—C3—C4—C5	−2.0 (4)	N1—C15—C16—C17	177.3 (3)
C8—C3—C4—S1	178.89 (19)	N1—C15—C20—C19	−177.4 (2)
C9—C1—N1—C2	73.2 (3)	O2—C2—C3—C4	−152.3 (3)
C9—C1—N1—C15	−110.4 (2)	O2—C2—C3—C8	26.3 (3)
C9—C1—S1—C4	−67.89 (18)	O2—C2—N1—C1	−175.5 (2)
C9—C1—S1—O1	−177.94 (17)	O2—C2—N1—C15	8.1 (4)
C9—C10—C11—C12	0.7 (4)	S1—C1—C9—C10	120.8 (2)
C10—C9—C14—C13	−1.4 (4)	S1—C1—C9—C14	−61.4 (3)
C10—C11—C12—C13	−0.8 (5)	S1—C1—N1—C2	−52.8 (3)
C11—C12—C13—C14	−0.1 (6)	S1—C1—N1—C15	123.56 (19)
C12—C13—C14—C9	1.3 (5)	S1—C4—C5—C6	−179.5 (2)
C14—C9—C10—C11	0.5 (4)		

### Hydrogen-bond geometry (Å, º)

**Table:**

*D*—H···*A*	*D*—H	H···*A*	*D*···*A*	*D*—H···*A*
C1—H1···O2^i^	0.98	2.31	3.240 (3)	157

Symmetry code: (i) −*x*+1/2, *y*−1/2, −*z*+1/2.
